# Smoking and tooth discolouration: findings from a national cross-sectional study

**DOI:** 10.1186/1471-2458-5-27

**Published:** 2005-03-24

**Authors:** Mhd N Alkhatib, Ruth D Holt, Raman Bedi

**Affiliations:** 1 Department of Dental Public Health, Guy's King's & St Thomas' Dental Institute, Caldecot Road, Denmark Hill Campus, London, SE5 9RW, UK; 2 International Centre for Excellence in Dentistry (ICED), Eastman Dent Inst, University College London, London, WC1XWD, UK

## Abstract

**Background:**

Smoking is a risk factor of a number of oral diseases; the extent to which tobacco products influence dental aesthetics has not been widely investigated. The aim of this study was to determine satisfaction with own tooth colour of smokers and non-smokers and to investigate whether smokers have higher levels of self-assessed tooth discolouration compared to non-smokers

**Methods:**

A cross sectional national study was conducted on sample of 6,000 UK adults. A total of 3,384 adults was interviewed. Smoking behaviour was recorded together with satisfaction with own tooth colour. Prevalence of perceived discolouration was measured by asking respondents to match their own tooth colour to one of a set of seven photographs of differing severities of discolouration.

**Results:**

Twenty eight percent of smokers reported having moderate and severe levels of tooth discolouration compared to 15% in non-smokers. As well as more often perceiving discolouration smokers were also more likely to be dissatisfied with their own tooth colour compared to non-smokers.

**Conclusion:**

The study provides further evidence of the negative impact of tobacco smoking on dental aesthetics in the general public. The evidence provided by the study may be of value in short interventions for smoking cessation in the dental setting.

## Background

In the last few decades there has been a substantial growth in strategies and measures to reduce smoking, particularly among young people. These have resulted in a decline in smoking prevalence in UK between the 1950's and the1990's [1]. Nevertheless, in the most recent survey of smoking Britain, more than a quarter of the UK adult and 10% of children said they were regular smokers [1,2].

Smoking is a major risk factor for general health. In the oral cavity it can lead to oral mucosal lesions, oral cancer, periodontal disease and consequent tooth loss [3-6]. However, perhaps the most visible and immediate dental manifestation seen by the public is tooth discolouration. Smokers' teeth tend to develop tobacco stains; these may be yellow, brown, dark brown or even black stains, the severity depending partly on duration and frequency of the habit. Tooth discolouration may therefore have a deleterious effect on individual's appearance, which in turn may result in social disadvantage for smokers.

In terms of efforts to encourage and support smoking cessation, it has been shown that when smokers are shown the adverse effect of smoking then they are more likely to quit than if other measures are used to motivate the same change [7]. In Finland, short intervention involving an exercise where adolescents identified stains on their teeth as a result of their smoking behaviour, helped to reduce the habit [8]. Labelling of cigarettes and tobacco carries mandatory health warnings. These are usually in the form of words, often against a white background and in marked contrast to the often seductive, design of the remainder of the packaging. The majority of warning labels on tobacco products are aimed at the more serious health problems of tobacco use; they are more often focused on lung cancer, impact of smoking on pregnancy, fertility or on general health. However, in the UK it was reported that smokers were not greatly influenced by the warring labels [9]. Very few used aesthetics or pictures in their health messages. In some tobacco products manufactured in Canada, actual pictures of tobacco staining are shown clearly on tobacco packs. The effectiveness of using images particularly those showing teeth, gums or lung diseases was tested in Canada. Results showed that three quarters of smokers recalled these images on tobacco products and reported that they believe that using these images is more effective than the commonly used worded warnings [10].

Aesthetics may be a significant supporting measure in the anti-smoking campaigns or interventions; this can be particularly important when targeting adolescents or females in general as appearance is more important to these two groups [11] who are often the desired target. Perhaps there is need of selecting the most effective warning message for the desired target.

In the United Kingdom there has been no national recording of the prevalence of tobacco staining. The aims of this study were firstly to investigate the prevalence of self-assessed discolouration in smokers and non-smokers, secondly to compare satisfaction with own tooth colour in the same two groups.

## Methods

The data collected here is part of a larger study investigating perception of dental fluorosis in the UK. Details have been published elsewhere (12). The office of national statistics omnibus survey was utilised to collect the data. A random stratified probability sample of UK adults was selected using the Postcode Address File (PAF). The study population included 6,000 addresses of adults aged 16 and above. The survey was carried out by 16 trained interviewers.

The questionnaire used in the study included a question about smoking behaviour (whether the respondent was a regular smoker or not) as part of socio-demographic and behavioural information gathered from the respondents. A set of six photographs showing varying severities of discolouration (a pair for each level) and one showing normal tooth colour was shown to respondents, who were asked to match their own tooth colour to the closest photograph in the set. Satisfaction with own tooth colour was measured on a five point scale which was then grouped into three point scale for analytical purposes. Classification of discolouration used in the photographs is described in detail in a separate paper (12). The questionnaire was piloted and tested for reliability and validity on samples of the public and professionals. Logistic regression was undertaken to calculate odds ratios and to adjust for potential confounding factors and descriptive analysis was carried out to provide frequency distribution. Data was analysed using the SPSS statistical package (version 11).

## Results

Three thousand nine hundred and fifty five of the 6,000 selected addresses were eligible and contactable. 3,384 adults agreed to take part in the study (representing a 69% response rate) and 3,215 provided information about tooth discolouration. Profile of the study sample is summarised in table 1. Almost three quarters 74% (2,387) of these were non-smokers; the remaining 26% (828) were regular smokers. Prevalence of tooth discolouration is presented in table 2. More than half of non-smokers (52%) reported themselves to have normal tooth colour whilst less than half (46%) of smokers reported the same. Moderate and severe discolourations were more prevalent in smokers.

**Table 1:** Profile of the study sample

**Sex**	Male	1500 (44.3%)
	Female	1884 (55.7%)
**Age**	16–34	957 (28.3%)
	35–54	1214 (35.9%)
	55+	1213 (35.8%)

**Education**	High education	748 (22.1%)
	Middle education	1304 (38.5%)
	Lower education	1332 (39.4)

**Income**	Average or higher than national average	1046 (30.9%)
	Lower than average	2338 (69.1%)

**Smoking**	Smoker	817 (24.1%)
	Non smoker	2567 (75.9%)

**Table 2:** Prevalence of tooth discolouration in smokers and non-smokers

**Tooth discolouration**	**Non-smokers N (%)**	**Smokers N (%)**
**Normal**	1236 (51.8%)	379 (45.8%)
**Mild**	774 (32.4%)	224 (27.1%)
**Moderate**	275 (11.5%)	149 (18.0%)
**Severe**	102 (4.3%)	76 (9.2%)

Results of the logistic regression of smoking and tooth discolouration are shown in table 3. The difference in the prevalence of mild tooth discolouration in smokers and non-smokers was not statistically significant (*P *> 0.05). However, in the case of moderate discolouration, smokers were more likely to report themselves to have discolouration of this severity [OR = 1.7 (1.4–2.2)] (*P *< 0.01), likelihood of having severe discolouration was even greater amongst smokers [OR = 2.4 (1.7–3.3)] (*P *< 0.01).

**Table 3:** Logistic regression of smoking and tooth discolouration

		***P *Value**	**OR**	95% CI**	
**Smokers**	Mild	*0.54*	0.94	0.78	1.13
	Moderate	0.00*	1.76	1.40	2.22
	Severe	0.00*	2.43	1.76	3.34

* Statistically significant at 0.05 level

** Confidence Interval

Age, sex and income were adjusted for in the regression. Non-smokers is the reference

Satisfaction with own tooth colour decreased with increasing severity of reported discolouration. Results are shown in table 4. Thirty percent of smokers were dissatisfied with their tooth colour compared to 15% of non-smokers. This difference was confirmed in the logistic regression where smokers were more likely to be dissatisfied compared to non-smokers [OR = 2.4 (2.0–-3.0)] (*P *< 0.01) (Table 5).

**Table 4:** Satisfaction with own tooth colour in smokers and non-smokers

	**Satisfied N (%)**	**Dissatisfied N (%)**	**Neither Satisfied nor dissatisfied N (%)**
**Smokers**	464 (56.1%)	250 (30.2%)	113 (13.7%)
**Non-smoker**	1678 (70.7%)	366 (15.3%)	334 (14.0%)

**Table 5:** Logistic regression of smoking and satisfaction with tooth colour

		***P *Value**	**OR**	95% CI**	
**Smokers**	Satisfied	0.00*	0.43	0.35	0.52
	Dissatisfied	0.00*	2.48	2.05	3.00
	Neither	*0.08*	1.23	0.97	1.55

## Discussion

Prevalence of self-assessed tooth discolouration in smokers was almost twice of that reported by non-smokers. However, this was true only for moderate and severe levels. Not all tooth discolourations may be attributed to smoking; excessive intake of fluoride in early childhood is another risk factor for tooth discolouration as a result of dental fluorosis. However, in the case of fluorosis, the likelihood of developing moderate or severe discolouration in a low fluoridated area such as UK is low so that where mild discolouration may be attributed to fluoride intake, more severe forms of discolouration may be attributed to smoking. Thus the pattern of perception may be logical.

Not only respondents who smoked were more likely to perceive discolouration they were also more likely to be dissatisfied with their appearance. Findings therefore suggest that smoking does have a negative impact on tooth colour and on perceived dental aesthetics. The potential for dental aesthetics to have more general effects such negative personal and social impacts has been shown by other researchers [13]

## Conclusion

The study has shown that smokers have higher prevalence of tooth discolouration than non-smokers as anticipated. Variations in the prevalence tooth discolouration were clearer in the case of more severe levels.

It has been reported that short interventions for smoking cessation are applicable by the dental team [8,14]. Information from this study highlighting the adverse cosmetic effect of smoking may provide a strong evidence base for strategies used for such interventions in primary care settings or in the dental practices. Use of pictures of teeth with tobacco staining as warning messages on tobacco products may be also worth consideration as it was very clear that smokers recognised the deleterious effect of smoking on their dental appearance.

Further research is needed to explore the impact of different types of tobacco products and other related factors such as duration and frequency of use on tooth colour.

## Authors' contributions

MNK and RB designed the study and produced the results, RH organised the analysis. MNK and RH have written the paper. All authors read and approved the final manuscript.

## Competing interests

The author(s) declare that they have no competing interests.

## Pre-publication history

The pre-publication history for this paper can be accessed here:


